# Supplementary material for: Flexible active-matrix micro-LED display with 1T-1FeMFET architecture featuring scaling-limit-free design
Source: Nat Commun. 2026 Mar 31;17:4628. doi: 10.1038/s41467-026-71182-9 (PMC13199476; doi:10.1038/s41467-026-71182-9)
Supplement: Supplementary file 1 — Supplementary Information [file 41467_2026_71182_MOESM1_ESM.pdf]

Supplementary Information

Flexible Active-Matrix Micro-LED Display with 1T-1FeMFET Architecture Featuring Scaling-Limit-Free Design

Tingrui Huang[1]†, Guangan Yang [2]†, Yimeng Sang[3]†, Fulin Zhuo[4], Mingming Liu[1], Gengyu Li[1], Heng Cheng[1], Zuoxu Yu[1], Zhiyuan Fu[1], Siyang Liu[1], Haoliang Shen[5], Zhihao Yu[2], Bin Liu[4], Xinran Wang[4, 5], Wangran Wu[1]*, Runxiao Shi[1]*, Zhe Zhuang[3]*, Weifeng Sun[1]*

[1]School of Integrated Circuits, National ASIC System Engineering Research Center, Southeast University, Nanjing 210096, China.

[2]College of Integrated Circuit Science and Engineering, Nanjing University of Posts and Telecommunications, Nanjing 210023, China.

[3]School of Integrated Circuits, Nanjing University 215163, Nanjing, China.

[4]School of Electronic Science and Engineering, Nanjing University, Nanjing 210093, China.

[5]Suzhou Laboratory, Suzhou 215004, China.

†These authors contributed equally: Tingrui Huang, Guangan Yang, Yimeng Sang.

*Correspondence to: wrwu@seu.edu.cn, icrshi@seu.edu.cn, zzhuang@nju.edu.cn, swffrog@seu.edu.cn.

This PDF file includes:

Supplementary Figures 1 to 19 (Pages S4-S14)

Supplementary Tables 1 to 2 (Pages S15-S16)

Supplementary References (Page S17-S18)

Supplementary Figure 1 The performance of FeCAP versus HZO thickness and PMA temperature.

Supplementary Figure 2 The *P*-*V* hysteresis loops during wake-up until 10^5 cycles.

Supplementary Figure 3 Polarization switching depends on the electric field across the HZO layer (E_{HZO}).

Supplementary Figure 4 The initialize-modulate-measure-verify procedure.

Supplementary Figure 5 Optical photograph of the PI film and μ-LED display.

Supplementary Figure 6 Detailed operating explanation of the ITO FeMFET.

Supplementary Figure 7 Transfer curves for the ITO TFTs with various *W*/*L*.

Supplementary Figure 8 Schematic illustration of charging trapping.

Supplementary Figure 9 Hysteresis transfer curves of ITO FeMFETs with various FeCAP areas (A_{Fe}).

Supplementary Figure 10 Hysteresis transfer curves of ITO FeMFETs with various channel dimensions (A_{De}).

Supplementary Figure 11 Definitions of specific current in transient and static testing.

Supplementary Figure 12 The output characteristics of ITO FeMFETs after programming and erasing pulses.

Supplementary Figure 13 Transient switching measurement on the ITO FeMFET.

Supplementary Figure 14 The endurance property of the ITO FeMFET.

Supplementary Figure 15 Voltage sequence and corresponding static characteristics of the 1T-1FeMFET pixel circuit.

Supplementary Figure 16 The electroluminescent spectra of the μ-LED before and after LLO from sapphire.

Supplementary Figure 17 On-wafer external quantum efficiency (EQE) of the μ-LED.

Supplementary Figure 18 The detailed fabrication process flow of the flexible active-matrix μ-LED display.

Supplementary Figure 19 *P*-*V* hysteresis loops expand with increasing PMA

temperature.

Supplementary Table 1. Benchmarks of $2P_r$ and maximum number of bending cycles of the flexible FeCAP reported in this work versus recently reported HZO-based FeCAPs.

Supplementary Table 2. Benchmarks of M.W. and I_{on}/I_{off} performance of the ITO FeMFET reported in this work versus recently reported FeFETs.

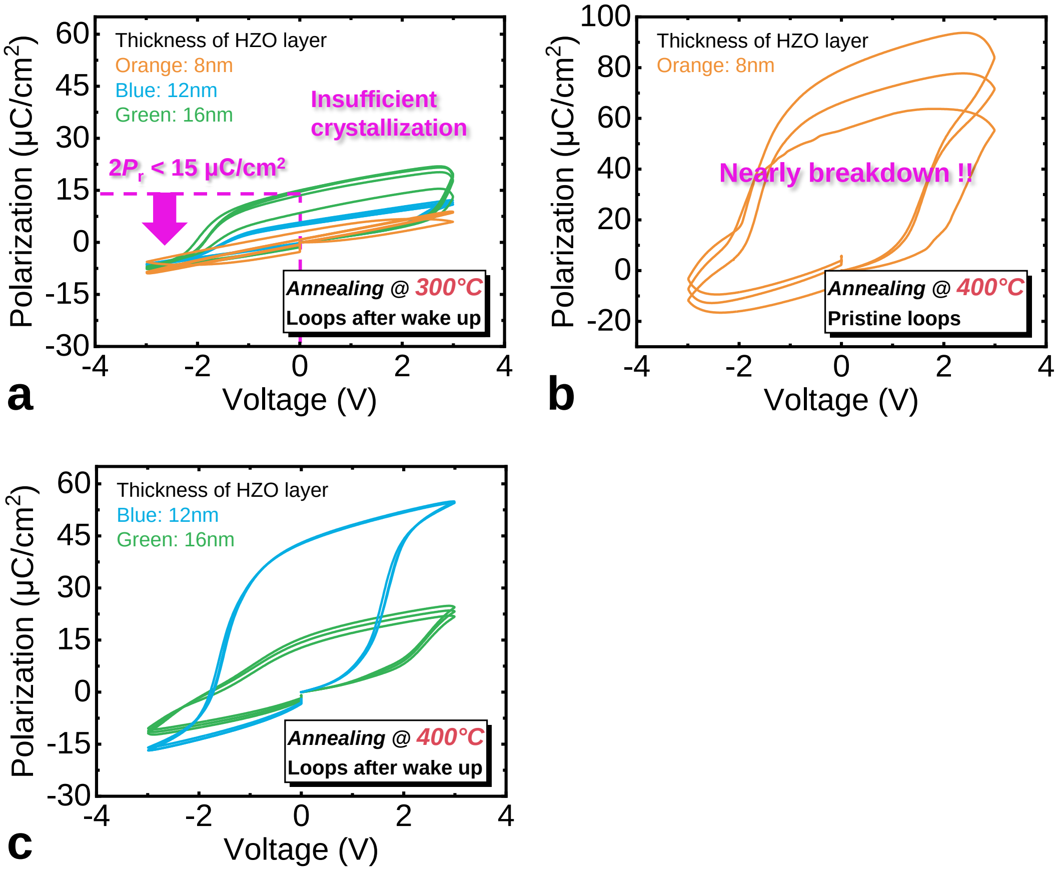

Supplementary Figure 1 | The performance of FeCAP versus HZO thickness and PMA temperature. a, *P*-*V* hysteresis loops of the FeCAPs with various HZO thickness after PMA at 300°C. **b,** *P*-*V* hysteresis loops of the FeCAPs with 8-nm-thick HZO after PMA at 400°C. **c,** *P*-*V* hysteresis loops of the FeCAPs with 12-nm and 16-nm-thick HZO after PMA at 400°C.

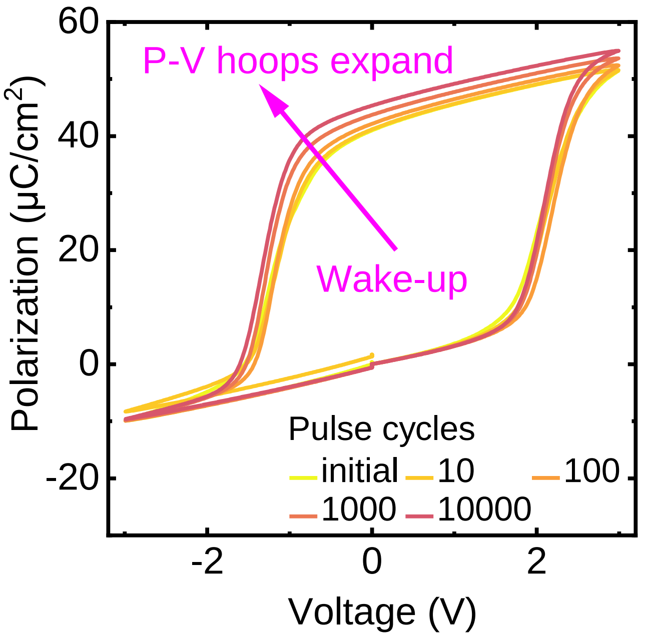

Supplementary Figure 2 | The *P*-*V* hysteresis loops during wake-up until 10^5 cycles.

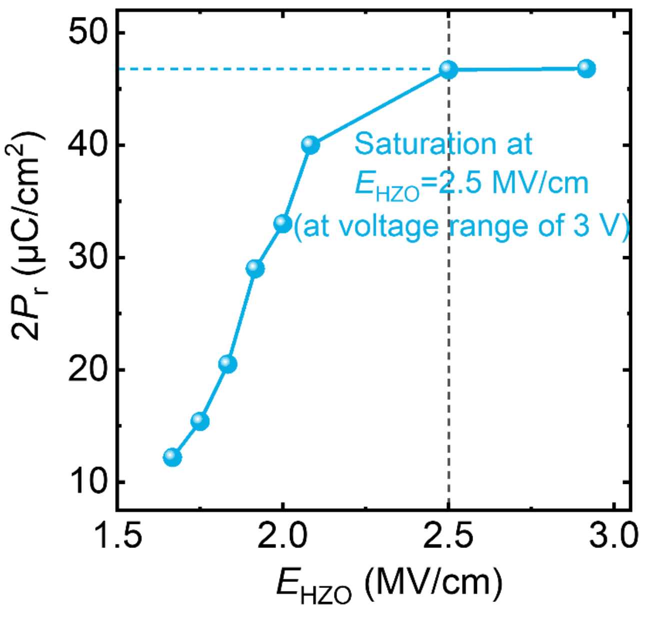

Supplementary Figure 3 | Polarization switching depends on the electric field across the HZO layer (E_{HZO}).

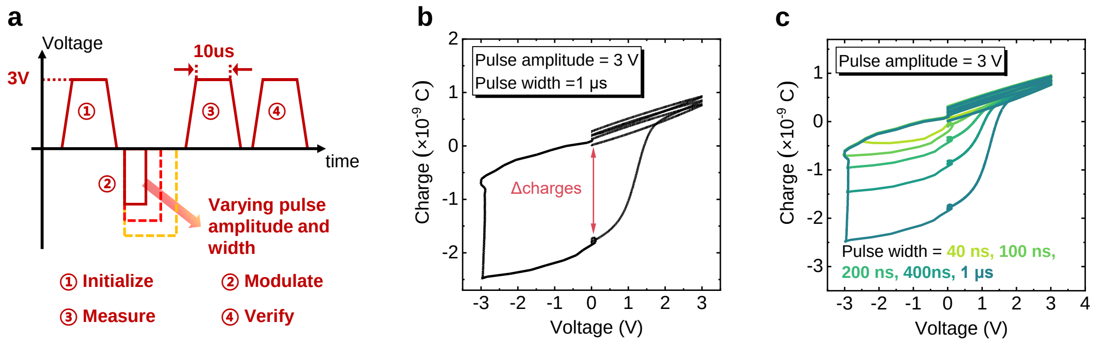

Supplementary Figure 4 | The initialize-modulate-measure-verify procedure. a, Voltage sequence of initialize-modulate-measure-verify procedure. By varying the amplitude and width of "Modulate" pulse (pulse 2), the polarization can be systematically modulated. **b,** Charges versus voltage at pulse amplitude of 3 V and pulse width of 1 μs. The partial polarization switching is calculated via the change of charges (Δcharges). **c,** Charges versus voltage at pulse amplitude of 3 V and under various pulse width. Δcharges increase as the "Modulate" pulse intensity increases.

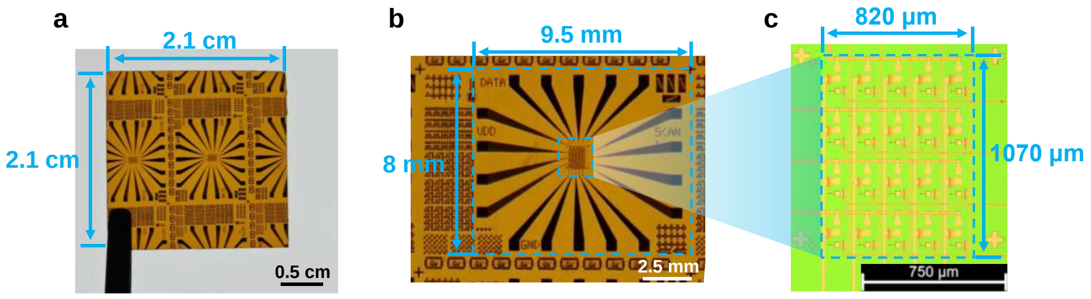

Supplementary Figure 5 | Optical photograph of the PI film and μ-LED display. a, Optical image of the PI film after laser-lifted-off (LLO). **b,** Optical image of the complete μ-LED display module with peripheral signal lines. **c,** Micrograph of the active μ-LED display array based on the 1T-1FeMFET pixel circuit.

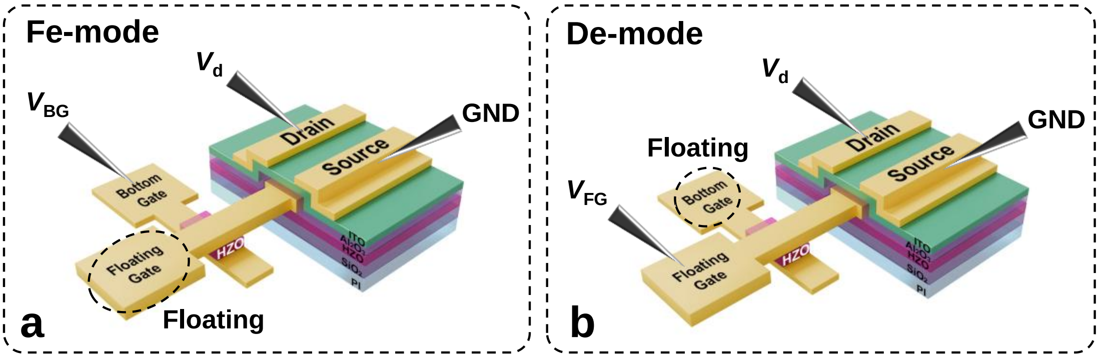

Supplementary Figure 6 | Detailed operating explanation of the ITO FeMFET. Detailed operating explanation of Fe-mode (**a**) and De-mode (**b**) for the proposed ITO FeMFET.

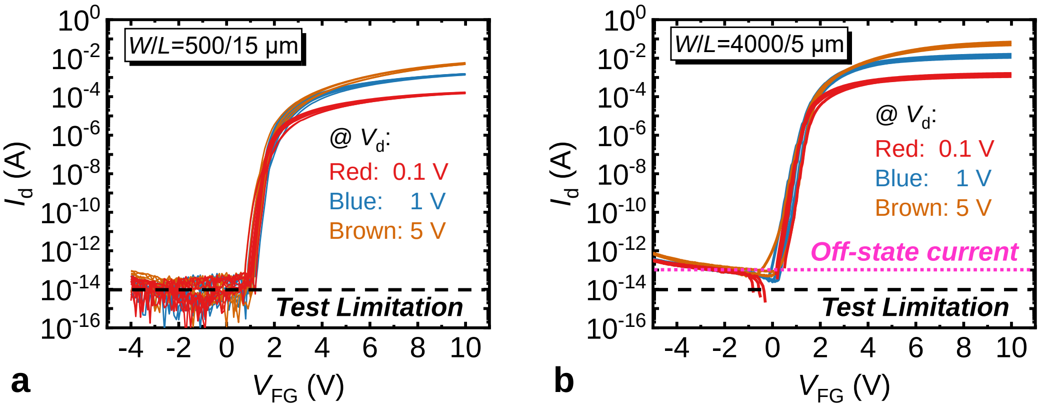

Supplementary Figure 7 | Transfer curves for the ITO TFTs with various *W*/*L*. Transfer curves for the ITO TFTs with *W*/*L* of 500 μm/15 μm (**a**) and 4000 μm/5 μm (**b**).

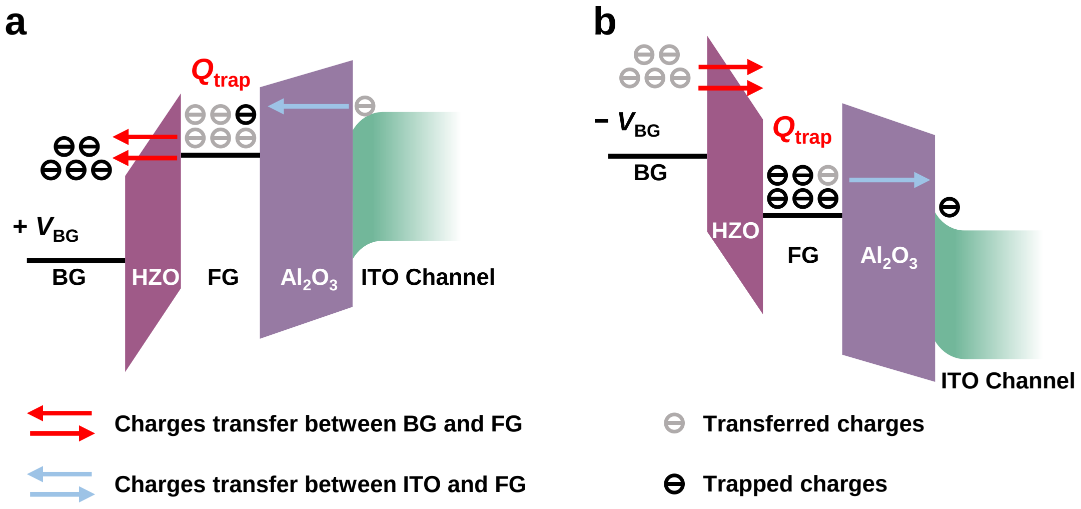

Supplementary Figure 8 | Schematic illustration of charging trapping. The energy band diagrams of the charge trapping in the FG under positive (**a**) and negative (**b**) applied voltage.

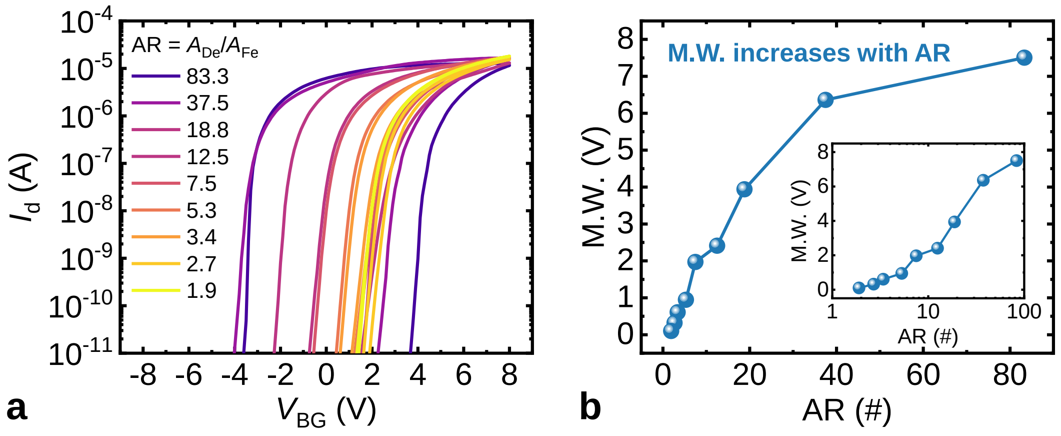

Supplementary Figure 9 | Hysteresis transfer curves of ITO FeMFETs with various FeCAP areas (A_{Fe}). Hysteresis transfer curves (**a**) and extracted M.W. (**b**) of the ITO FeMFET as a function of the AR, with various FeCAP areas (A_{Fe}) and a fixed channel dimension (A_{De}) of 750 μm^2.

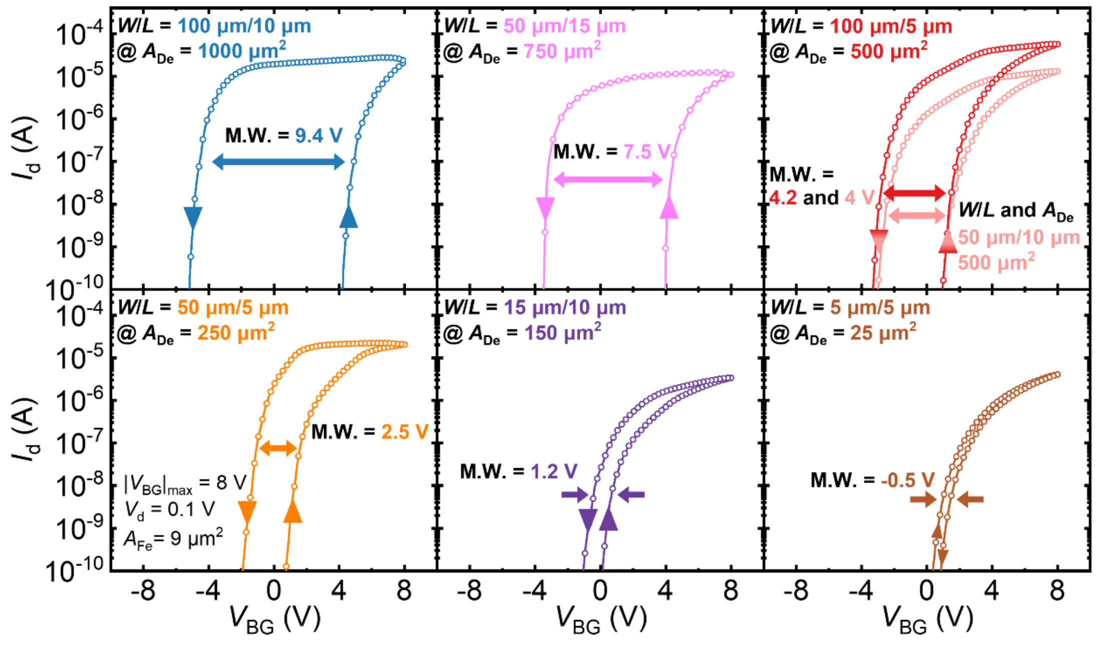

Supplementary Figure 10 | Hysteresis transfer curves of ITO FeMFETs with various channel dimensions (A_{De}).

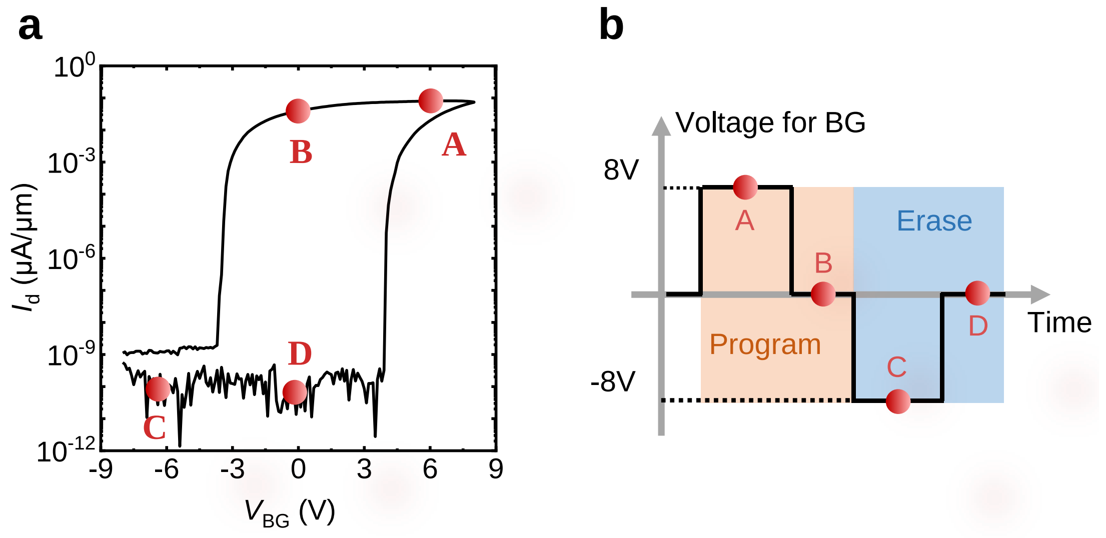

Supplementary Figure 11 | Definitions of specific current in transient and static testing. a, I_d (at V_{BG} = 0 V) for the backward (or forward) sweep transfer curve is also denoted as I_{on} (or I_{off}), corresponding to point B (or D) of the hysteresis transfer curves. **b,** I_d (at V_{BG} = 0 V) following a positive (or negative) pulse is designated as I_{on} (or I_{off}), corresponding to point B (or D) in the voltage sequence.

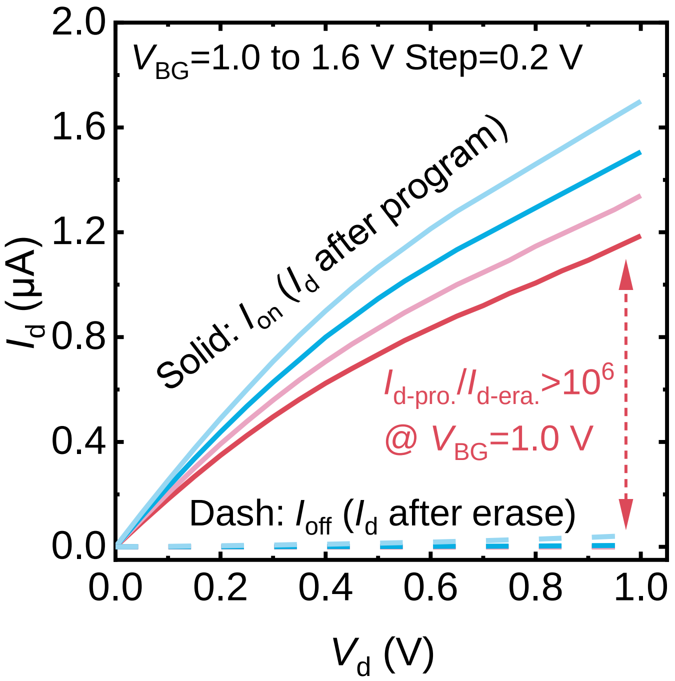

Supplementary Figure 12 | The output characteristics of ITO FeMFETs after programming and erasing pulses.

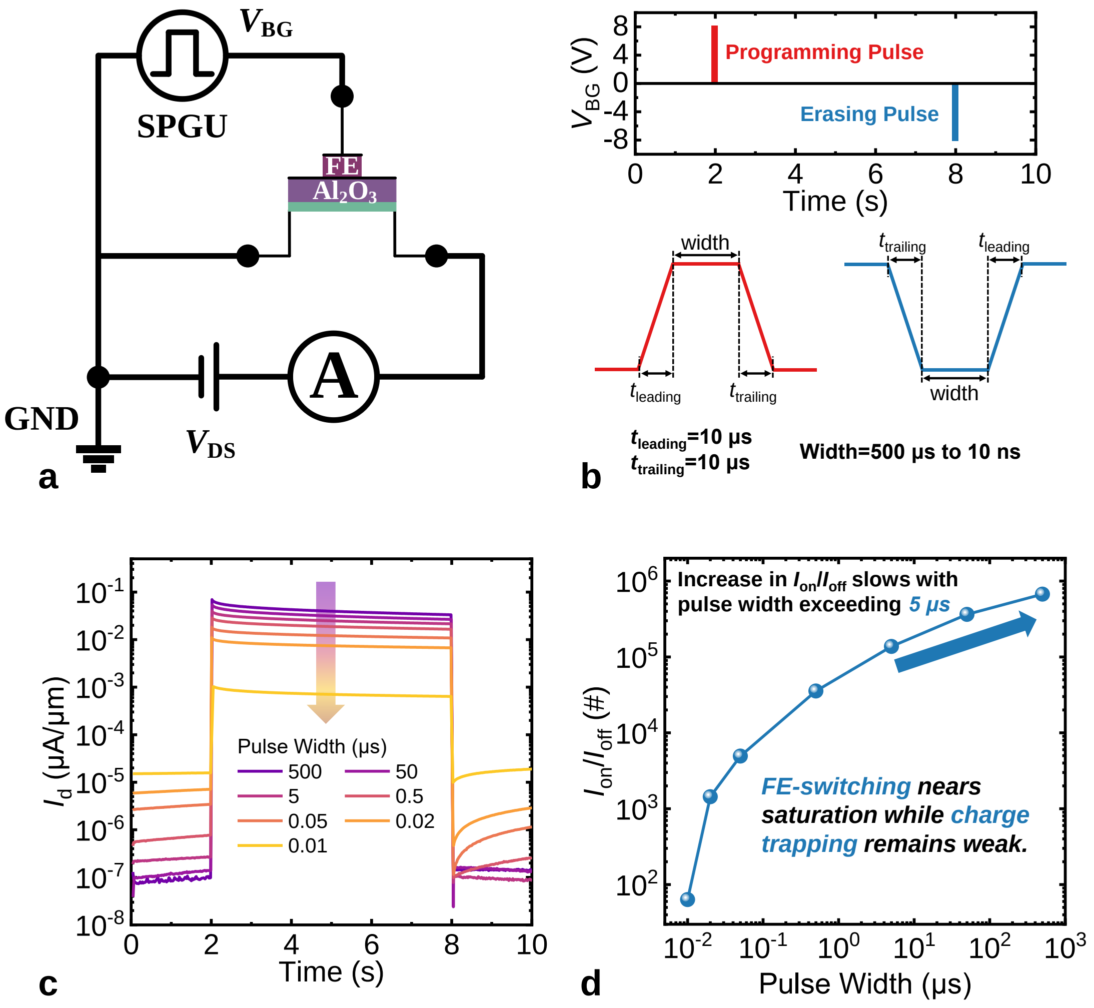

Supplementary Figure 13 | Transient switching measurement on the ITO FeMFET. Schematic of the transient switching measurement (**a**) for the ITO FeMFET and the measuring voltage sequence (**b**). Response of I_d (**c**) and I_{on}/I_{off} (**d**) for the ITO FeMFET to programming and erasing pulses with pulse widths ranging from 500 μs to 10 ns.

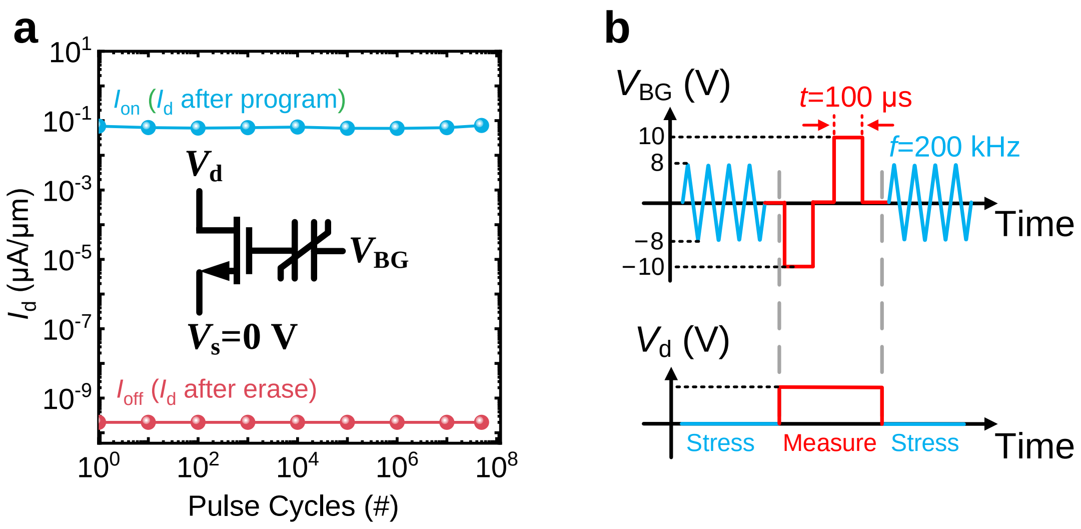

Supplementary Figure 14 | The endurance property of the ITO FeMTFT. a, The endurance property of the ITO FeMFET. Inset shows the testing scheme. **b,** The voltage sequence scheme for stress and measurement.

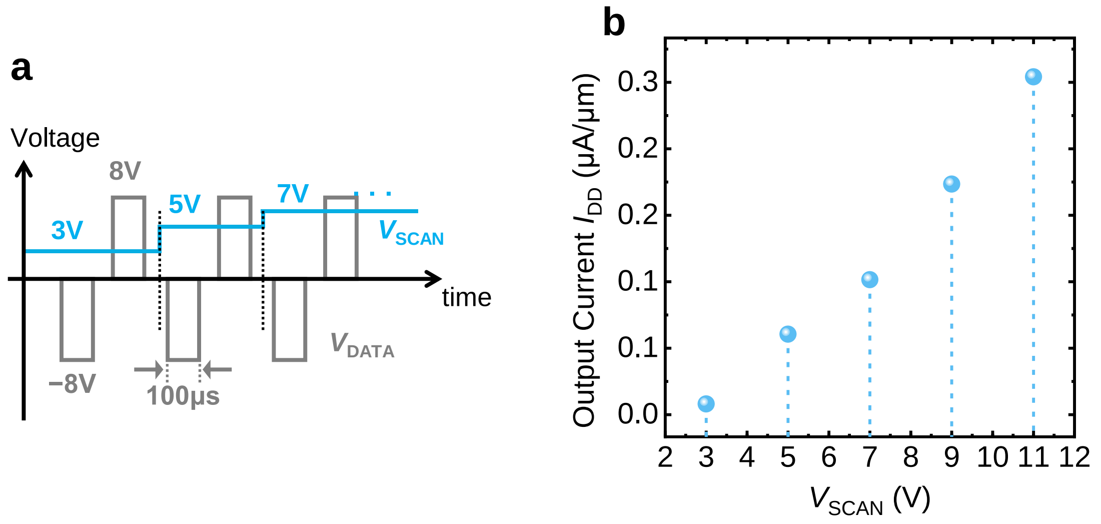

Supplementary Figure 15 | Voltage sequence and corresponding static characteristics of the 1T-1FeMFET pixel circuit. a, Voltage sequence for static characteristics. **b,** Static characteristics of the 1T-1FeMFET pixel circuit.

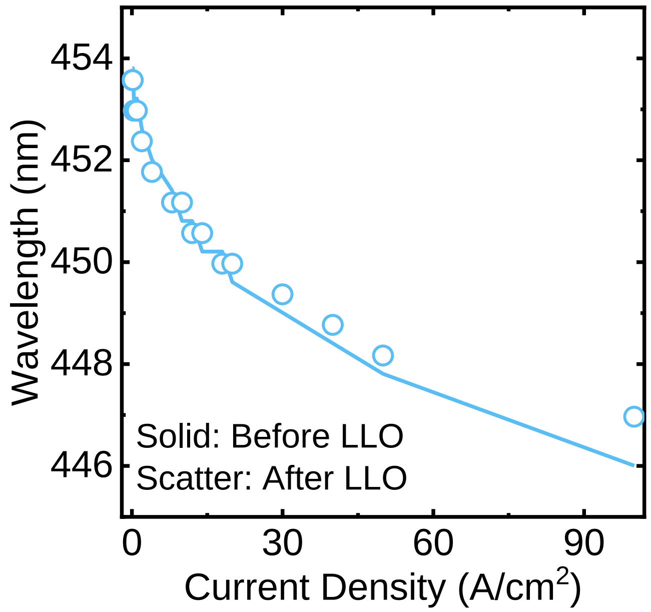

Supplementary Figure 16 | The electroluminescent spectra of the μ-LED before and after LLO from sapphire.

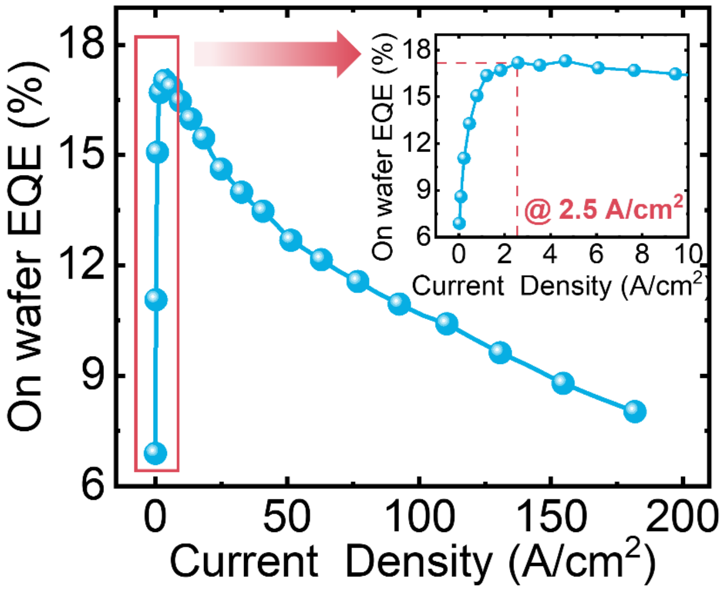

Supplementary Figure 17 | On-wafer external quantum efficiency (EQE) of the μ-LED.

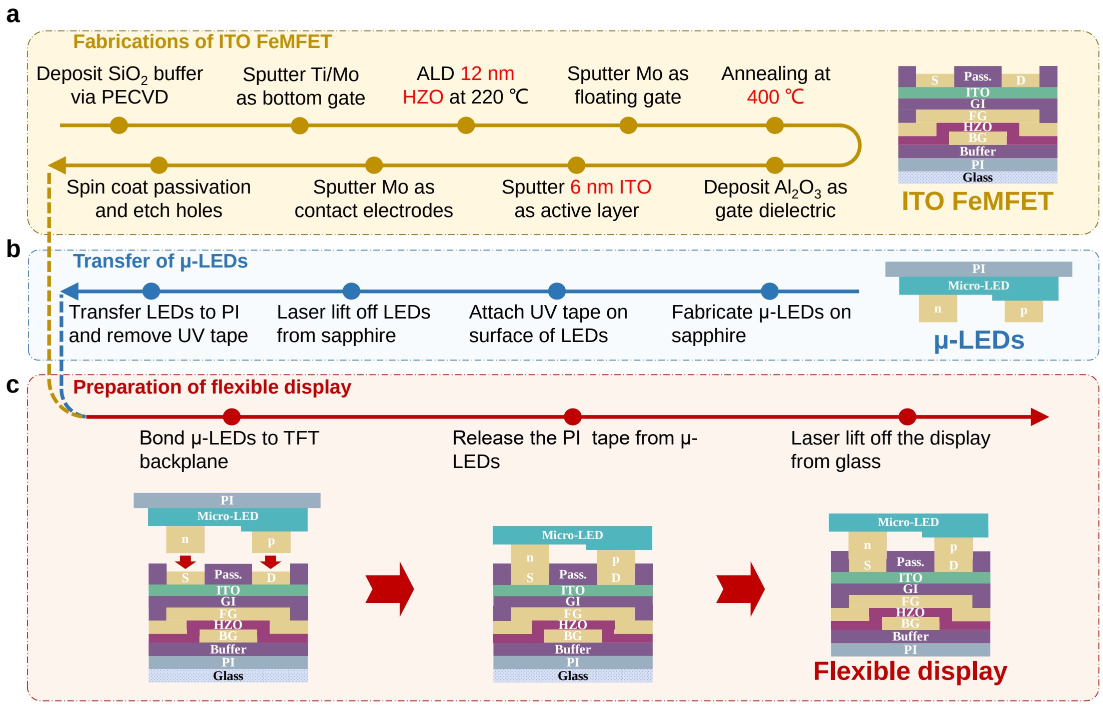

Supplementary Figure 18 | The detailed fabrication process flow of the flexible active-matrix μ-LED display. a, Fabrication process flow of the ITO FeMFET. **b,** Transfer of μ-LEDs. **c,** Preparation of flexible display.

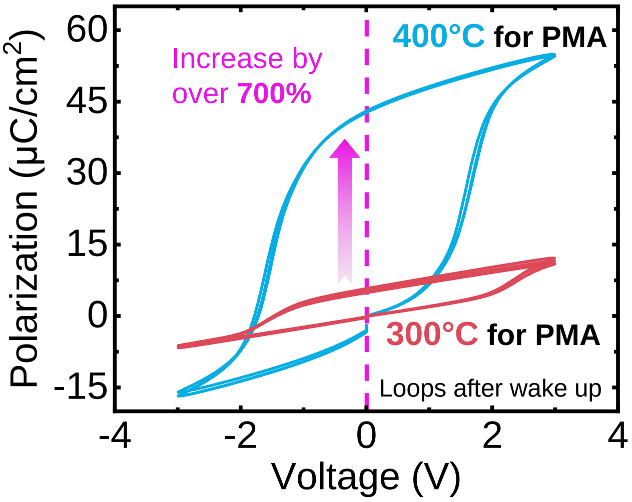

Supplementary Figure 19 | *P-V* hysteresis loops expand with increasing PMA temperature.

Supplementary Table 1 | Benchmarks of $2P_r$ and maximum number of bending cycles of the flexible FeCAP reported in this work versus recently reported HZO-based FeCAPs.

Structure	FE	Thickness of FE (nm)	Material of flexible substrate	Process temperature (°C)	$2P_r$ (μC/cm²)	Max. number of bending cycles	$r_{bending}$ during cycling (mm)	year	Ref.
TiN/WO_x/HZO/ Wo_x/TiN	HZO	10	Polyimide	700	37.6	10000	2	2025	1
TiN/HZO/TiN	HZO	20	Mica	500	32.5	1000	6	2020	2
W/HZO/W	HZO	10	Polyethylene terephthalate	600	31	1000	9	2022	3
TaN/HZO/TaN	HZO	18	Mica	500	18	1000	7.5	2019	4
TiN/HZO/TiN	HZO	10	Polyimide	400	21	5000	2	2021	5
W/HZO/W	HZO	10	Silicon	500	29.5	6000	12	2023	6
IGZO/HZO/Mo	HZO	35	Polyimide	300	21	10000	5	2025	7
**Mo/HZO/Mo**	**HZO**	**12**	**Polyimide**	**400**	**47**	**10000**	**4**	**2025**	**This work**

Supplementary Table 2 | Benchmarks of M.W. and I_{on}/I_{off} performance of the ITO FeMFET reported in this work versus recently reported FeFETs.

Channel	FE	M.W. (V)	Thickness of FE (nm)	Normalized M.W. (V/nm)	*W/L* and Area (μm/μm, $μm^2$)	I_{on}/I_{off}	year	Ref.
ITO	HZO	2.78	11.4	0.24	50/5, 250	10^8	2023	8
MoS_2	HZO	9.5	16	0.59	/	10^7	2023	9
InAs	HZO	1.53	12	0.125	/	10^4	2022	10
IWO	HZO	2	10	0.2	20/0.25, 5	10^5	2022	11
$InZnO_x$	HZO	3.84	24	0.16	/	10^5	2023	12
IZTO	HZO	2	32	0.063	350/15, 5250	10^5	2021	13
In_2O_3	HZO	2.2	10	0.22	1.5/1.5, 2.25	10^7	2021	14
ITO/IGZO	HZO	2.1	10	0.21	0.5/0.04, 0.02	10^7	2022	15
IWO	HZO	1.45	10	0.145	20/0.25, 5	10^5	2021	16
		**9.4**		**0.78**	**100/10, 1000**	$\mathbf{10^9}$		Target Device
		**7.5**		**0.63**	**50/15, 750**	$\mathbf{4\times10^8}$		
		**4.2**		**0.35**	**100/5, 500**	$\mathbf{5\times10^8}$		
**ITO**	**HZO**	**4**	**12**	**0.33**	**50/10, 500**	$\mathbf{10^8}$	**2025**	**This work**
		**2.5**		**0.21**	**50/5, 250**	$\mathbf{10^8}$		
		**1.2**		**0.1**	**15/10, 150**	$\mathbf{10^6}$		
		**0.5 (CW)**		**0.04 (CW)**	**5/5, 25**	**/**		

Supplementary References

1. Zhao, C., Wang, H., Gu, X., Zhang, W. & Li, Y. Ultrathin WO interfacial layer improving the ferroelectricity and endurance of $Hf_{0.5}Zr_{0.5}O_2$ thin films on polyimide. *Journal of Materiomics* **11** (2025).
2. Liu, H. *et al.* Flexible Quasi-van der Waals Ferroelectric Hafnium-Based Oxide for Integrated High-Performance Nonvolatile Memory. *Advanced Science* **7** (2020).
3. Liu, B. *et al.* Excellent HZO ferroelectric thin films on flexible PET substrate. *Journal of Alloys and Compounds* **919** (2022).
4. Xiao, W. *et al.* Thermally Stable and Radiation Hard Ferroelectric $Hf_{0.5}Zr_{0.5}O_2$ Thin Films on Muscovite Mica for Flexible Nonvolatile Memory Applications. *ACS Applied Electronic Materials* **1**, 919-927 (2019).
5. Chen, Y. *et al.* Flexible $Hf_{0.5}Zr_{0.5}O_2$ ferroelectric thin films on polyimide with improved ferroelectricity and high flexibility. *Nano Research* **15**, 2913-2918 (2021).
6. Xie, X. *et al.* The Excellent Bending Limit of a Flexible Si-Based $Hf_{0.5}Zr_{0.5}O_2$ Ferroelectric Capacitor with an Al Buffer Layer. *Electronics* **13** (2023).
7. Rho, H. Y. *et al.* Plasma-irradiated hafnia ferroelectrics for high-performance flexible thin film transistors. *Materials Today Nano* **29** (2025).
8. Li, Q. *et al.* High-performance ferroelectric field-effect transistors with ultra-thin indium tin oxide channels for flexible and transparent electronics. *Nature Communications* **15** (2024).
9. Ning, H. *et al.* An in-memory computing architecture based on a duplex two-dimensional material structure for in situ machine learning. *Nature Nanotechnology* **18**, 493-500 (2023).
10. Aabrar, K. A. *et al.* BEOL-Compatible Superlattice FEFET Analog Synapse With Improved Linearity and Symmetry of Weight Update. *IEEE Transactions on Electron Devices* **69**, 2094-2100 (2022).
11. Kim, I.-J., Kim, M.-K. & Lee, J.-S. Design Strategy to Improve Memory Window in Ferroelectric Transistors With Oxide Semiconductor Channel. *IEEE Electron Device Letters* **44**, 249-252 (2023).
12. Kim, M.-K., Kim, I.-J. & Lee, J.-S. Oxide semiconductor-based ferroelectric thin-film transistors for advanced neuromorphic computing. *Applied Physics Letters* **118** (2021).
13. Lin, Z. *et al.* High-Peformance BEOL-Compatible Atomic-Layer-Deposited In_2O_3 Fe-FETs Enabled by Channel Length Scaling down to 7 nm: Achieving Performance Enhancement with Large Memory Window of 2.2 V, Long Retention > 10 years and High Endurance > 10^8 Cycles. *2021 IEEE International Electron Devices Meeting (IEDM)* (2021).
14. Chen, C.-K. *et al.* First Demonstration of Ultra-low Dit Top-Gated Ferroelectric Oxide-Semiconductor Memtransistor with Record Performance by Channel Defect Self-Compensation Effect for BEOL-Compatible Non-Volatile Logic Switch. *2022 International Electron Devices Meeting (IEDM)* (2022).
15. Aabrar, K. A. *et al.* BEOL Compatible Superlattice FerroFET-based High

Precision Analog Weight Cell with Superior Linearity and Symmetry. *2021 IEEE International Electron Devices Meeting (IEDM)* (2021).
16. Wang, K. *et al.* Investigation of LTPS and a-Si TFT pixel circuit for micro-light-emitting-triode with current gain. *Microelectronics Journal* **158** (2025).
